# A Pilot Study of Motivational Interviewing Training in a Virtual World

**DOI:** 10.2196/jmir.1825

**Published:** 2011-09-26

**Authors:** Suzanne Mitchell, Robin Heyden, Neil Heyden, Paul Schroy, Stephen Andrew, Ekaterina Sadikova, John Wiecha

**Affiliations:** ^1^Boston University School of MedicineDepartment of Family MedicineBoston, MAUnited States; ^2^Education ConsultantWellesley, MAUnited States; ^3^Educational ConsultantWellesley, MAUnited States; ^4^Boston University School of MedicineDepartment of GastroenterologyBoston, MAUnited States; ^5^Health Education and Training InstitutePortland, MEUnited States

**Keywords:** Medical education, continuing medical education, computer-assisted instruction, computer-aided instruction, distance education, computer simulation, patient simulation, motivational interviewing, virtual world

## Abstract

**Background:**

Motivational interviewing (MI) is an evidence-based, patient-centered counseling strategy proven to support patients seeking health behavior change. Yet the time and travel commitment for MI training is often a barrier to the adoption of MI by health care professionals. Virtual worlds such as Second Life (SL) are rapidly becoming part of the educational technology landscape and offer not only the potential to improve access to MI training but also to deepen the MI training experience through the use of immersive online environments. Despite SL’s potential for medical education applications, little work is published studying its use for this purpose and still less is known of educational outcomes for physician training in MI using a virtual-world platform.

**Objective:**

Our aims were to (1) explore the feasibility, acceptability, and effectiveness of a virtual-world platform for delivering MI training designed for physicians and (2) pilot test instructional designs using SL for MI training.

**Methods:**

We designed and pilot tested an MI training program in the SL virtual world. We trained and enrolled 13 primary care physicians in a two-session, interactive program in SL on the use of MI for counseling patients about colorectal cancer screening. We measured self-reported changes in confidence and clinical practice patterns for counseling on colorectal cancer screening, and acceptability of the virtual-world learning environment and the MI instructional design. Effectiveness of the MI training was assessed by coding and scoring tape-recorded interviews with a blinded mock patient conducted pre- and post-training.

**Results:**

A total of 13 physicians completed the training. Acceptability ratings for the MI training ranged from 4.1 to 4.7 on a 5-point scale. The SL learning environment was also highly rated, with 77% (n = 10) of the doctors reporting SL to be an effective educational medium. Learners’ confidence and clinical practice patterns for colorectal cancer screening improved after training. Pre- to post-training mean confidence scores for the ability to elicit and address barriers to colorectal cancer screening (4.5 to 6.2, *P* = .004) and knowledge of decision-making psychology (4.5 to 5.7, *P* = .02) and behavior change psychology (4.9 to 6.2, *P* = .02) increased significantly. Global MI skills scores increased significantly and component scores for the MI skills also increased, with statistically significant improvements in 4 of the 5 component skills: empathy (3.12 to 3.85, *P* = .001), autonomy (3.07 to 3.85, *P* < .001), collaboration (2.88 to 3.46, *P* = .02), and evocative response (2.80 to 3.61, *P* = .008).

**Conclusions:**

The results of this pilot study suggest that virtual worlds offer the potential for a new medical education pedagogy that will enhance learning outcomes for patient-centered communication skills training.

## Introduction

Patient-centered clinical skills, supported by versatile use of technology, enhance the clinical effectiveness of health care professionals. A cornerstone of effective health care is patient-centered communication and health behavior counseling, and the most widely studied approach is motivational interviewing (MI). Yet the intensive time and resource commitments for standard MI training often present barriers to the adoption of MI by health care professionals. Recently, however, the use of novel educational technologies such as virtual worlds has been showing great promise for overcoming access barriers to practical professional development training, such as is required for learning MI, and deepening the MI learning experience.

The popularity and scope of Web-based medical education curricula and continuing medical education (CME) programs has increased dramatically in recent years, but virtual-world venues are still not mainstreamed for these purposes [1-3]. Virtual worlds are rapidly becoming part of the general educational landscape and have untapped potential for meeting the unique and practical demands of medical education. Second Life (SL) is one of the best known virtual worlds, with over 300 colleges and universities staging regular events, seminars, and workshops in SL [4]. Most of these are education and awareness locations featuring kiosks and visual displays, health videos, slideshows, clinical skills simulation exercises, and Web links [5-11]. The advantages of a virtual-world venue such as SL for medical education include not only the convenience of a virtual-world venue that can be accessed from any physical location with an available computer, but also the immersive and 3-dimensionally realistic environment that creates a potentially richer learning experience than standard Web-based training courses.

What is SL? First launched in 2003, SL is an example of an immersive, 3-dimensional environment that supports a high level of social networking and interaction with information. Individuals can enter SL free of charge as avatars. In the SL virtual world, vast opportunities exist for student interaction, intense engagement, scripted immersive experiences, simulations, role-playing, and constructivist learning. The anonymity afforded by the avatar appears to lead to less inhibition and greater interaction. In addition, the greater sense of “presence” in a virtual world positively influences group process and cohesiveness, as well as engagement and attention [12]. We chose SL for our pilot study because it is the most widely used virtual-world platform, there is no access charge, and we had recent success conducting a CME event in SL on diabetes care management [13]. At any given time, 30,000 to 60,000 users are logged into SL. As of January 2009, there were 15 million registered SL accounts [14].

Little is known about the educational effectiveness of health professional training in SL or other virtual worlds, aside from our own published report in 2010 [13]. We published findings in the *Journal of Medical Internet Research* of our pilot study of a CME course conducted in SL on diabetes treatment. In our prior study, we focused on training physicians in a disease management skill. In the current study, we report findings from a new study whereby we explored the use of a virtual environment for training clinicians in MI counseling for colorectal cancer screening.

The purpose of this project was to explore the potential use of the SL virtual-world platform for training physicians in MI. The objectives of this pilot study were to (1) explore the feasibility, acceptability, and effectiveness of a virtual-world platform for delivering MI training designed for physicians, (2) pilot test instructional designs using SL for MI training, and (3) measure participant learning outcomes and feedback.

## Methods

### Study Sample and Recruitment

We targeted primary care residents and practicing physicians, although being a primary care practitioner was not required for participation. Participants were recruited from two family medicine listservs and from prior participants in our online courses. To be eligible for the pilot study, participants needed to communicate in English, be available on the dates of the synchronous SL sessions, and have computer equipment and capacity to support the SL platform. Given the anticipated time commitment, including training and pre- and post-training surveys, participants were offered an honorarium for completion of all activities. Since this was a research study and not an accredited CME program, no CME credits were provided. All participants took part in the experimental condition. Approval was obtained from Boston University School of Medicine institutional review board.

### Instructional and Study Design

The investigators commissioned an expert MI trainer to provide the session content advice and coach role-play sessions. The instructional emphasis was to understand the conceptual framework underpinning MI and to learn the MI health counseling approach through practice with feedback and coaching, based on available evidence on effective MI training approaches [15]. We focused on two specific MI skills, *developing empathic partnership* and *eliciting change talk,* because of the evidence correlating behavior change with these counseling behaviors [16]. Empathic partnership represents the global spirit of MI and is the process whereby the clinician establishes understanding, cultivates trust with the patient, and creates a safe haven wherein the patient explores his or her ambivalence about undertaking behavior change actions. Eliciting change talk involves the process of encouraging patients to discuss and describe a desire and vision for the specific behavior change. Change talk is considered the precursor step toward taking action [17].

**Figure 1:**
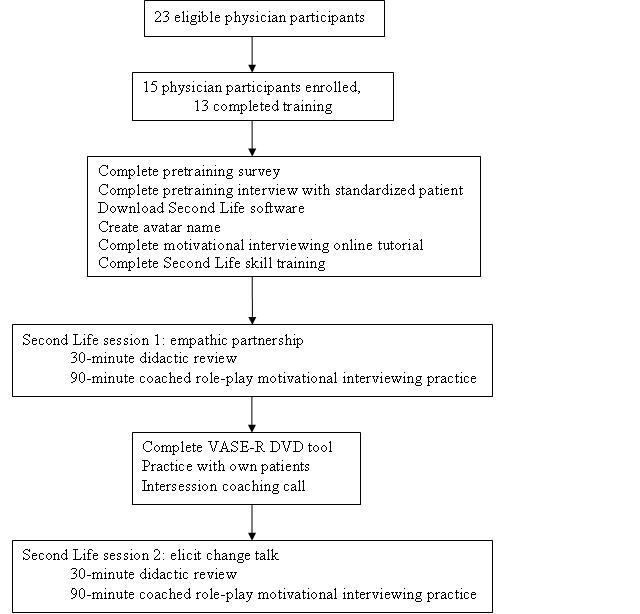
Recruitment and instructional design (VASE-R = video assessment of simulated encounters-revised).

We applied mixed educational approaches to train primary care physicians in MI based on evidence of best training practices (see Figure 1) [15]. In preparation for each SL event, all study participants completed a 1-hour online tutorial in MI to gain a fundamental understanding of the MI philosophy and underlying conceptual model. For the in-world events we modified two existing MI slide decks typically used for live trainings, to support two 30-minute reviews of didactic information focused on *developing empathic partnership* and *eliciting change talk*. We modified the presentation to allow opportunities for interaction between the trainer and the learners using local text chat and other activity afforded by the virtual world such as a simulated visual element to leverage the unique capabilities of SL. In the second SL session, we also added a model MI interview after the didactic presentation, prior to role-play exercises. The interview was conducted by the MI trainer with one of the standardized patients to help demonstrate MI skills in a health counseling session. We developed two standardized patient scripts for use interchangeably by the standardized patients for mock interviews. The scripts focused on a discussion of colorectal cancer screening with a patient who is ambivalent about undergoing screening. The study participants were asked to engage the standardized patient in a discussion about colorectal cancer screening using the principles of MI.

Following the didactic review and model interview, we engaged in practical skills development. To enhance the adoption of new communication skills, we employed a practical skill-building approach using role-play with standardized patients. To make this feasible, we split the learners into two small groups of 6–7 persons each and teleported them to two separate platforms, each with an MI coach and a standardized patient. For the remaining 75–90 minutes, each participating physician took a turn interviewing and counseling the standardized patient on colorectal cancer screening and received immediate feedback from the MI coach and other study participants. Learners also engaged in active observation of peer participants’ role-play experiences, feedback, and coaching, providing text chat feedback and commentary for one another.

Between sessions, we encouraged participants to practice the MI skills with their own patients. We also asked participants to complete the video assessment of simulated encounters-revised (VASE-R) DVD-based video assessment tool, developed by MI researchers at the University of Washington. The 18-item instrument includes three video-based vignettes, in which actors portray patients, with each vignette followed by questions that prompt learners to write responses that are then scored against MI standards [18]. The participants completed online answer sheets, which were scored by an independent and blinded MI expert, and we furnished results to participants as feedback. In addition, each participant received an individual coaching session by telephone with an expert MI coach. These coaching sessions were approximately 15 minutes in length and involved positive reinforcement, trouble-shooting of problem situations, and clarification of concepts if requested by the learner. In total, the training was designed to be completed in 8–10 hours of learners’ time.

### The Virtual-World Venue

**Figure 2:**
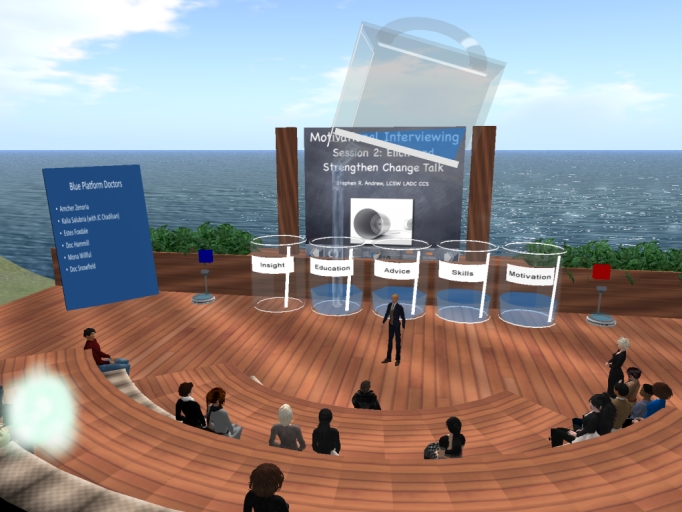
The virtual-world venue for motivational interviewing in Second Life training.

We started with an existing Boston University School of Medicine SL build (or “sim”) constructed on a private, virtual island owned and developed by Boston University School of Medicine for an earlier joint project between the School and the World Health Organization [19]. Ownership of the island allowed the developers to control access and thus provide security and privacy for the attending physicians. If an SL venue is not private, there is a risk of random avatars potentially disrupting an event. Further details on the virtual-world venue are reported elsewhere [13] (see Figure 2).

The participating physicians were on their own computers in their homes or offices across the United States while the investigators and the speaker conducted the seminar in the same physical location. This allowed easy communication between the study staff during the event and convenient technical support for the speaker during the event. As insurance against sound problems (one of the more often encountered SL technical issues), Skype names were collected from all participants in advance so that a Skype conference call could be placed between the speaker’s location and any participants experiencing sound problems [20]. During the two in-world events we had two technical support people available to help solve issues as they arose. Typical technical problems experienced by the participants included sound problems (cannot hear, cannot speak, hearing an echo, forgot to bring headphones), entering SL from a different computer and encountering firewall access problems, and trouble adjusting their camera view to read the slides. The seminar speaker was coached in one-on-one sessions conducted by phone. We conducted two dress rehearsals prior to the live SL training.

### Training in SL

Following eligibility screening and enrollment, each participant was given information on how to access the online SL tutorials. The tutorials were constructed based on an essential skills checklist required for participation in the planned activities. Required SL skills included moving the avatar (walking, flying, or sitting), using camera controls for viewing, communicating using text chat, talking, and instant messaging, and troubleshooting technical problems. Participants were instructed to work their way through the online tutorials on their own [21]. If they had questions or experienced problems, they could email for help or telephone during announced “office hours.” Prior to the first in-world event, each study participant took part in a short, scheduled skills assessment in order to determine their readiness for the training. Any problems or questions were addressed at that time.

### Outcome Measures

We assessed feasibility and acceptability of recruitment, the instructional design, and the virtual-world learning environment using a Web-based self-report questionnaire. We measured limited effectiveness of the MI training by first comparing changes in pre- and post-training responses to a 16-item questionnaire exploring learners’ self-reported confidence and clinical practice related to colorectal cancer screening. We measured MI skills proficiency by comparing the pre- and post-training scores from coded audiotaped mock interviews with a standardized patient. The scores were determined by a blinded, independent coder using the Motivational Interviewing Treatment Integrity, version 3.0 coding tool [22]. A difference of 1 on a 5-point scale between pre- and post-training scores was considered a clinically significant change in MI skills proficiency and was used for statistical testing as the relevant effect size [15]. According to this coding instrument, a global clinician score of 3.5 was used as the threshold score for proficiency and 4.0 indicated MI competency, out of a total possible score of 5. We calculated the mean scores for the core components and compared the mean global scores using the Wilcoxon signed rank test.

## Results

### Recruitment and Characteristics of Study Sample

Initially, 23 physicians expressed interest in the program. Following screening for eligibility, 15 of the original 23 participants enrolled in the study and 13 completed the training. After the first session, 2 enrolled participants dropped out due to conflicts with the workshop dates. The final sample comprised 2 male physicians and 11 female physicians, residing in 5 different states (Pennsylvania, Kansas, Massachusetts, Illinois, and New York). All 13 participants specialized in family medicine. Of the 13 participants, 10 had an average of 9 years of postresidency clinical practice experience (3 of the 13 are still enrolled in a residency program) and 5 are in academic practice. The majority of the participants (10/13, 77%) indicated they work in an environment with more than 10 other doctors, while 2 said they work with 4–6 doctors and 1 worked alone. Participants reported spending between 0 and 10 hours dedicated to the study training, with 4 participants spending more than 10 hours, 3 spending 6–10 hours, 3 spending 1–5 hours, and 3 spending 0 hours.

About a third (5/13, 38%) of the participants considered themselves “expert Internet users,” 62% (8/13) were steady users, and the majority (12/13, 92%) reported being “very comfortable” or “moderately comfortable” with using the computer. Only 2 participants said that they had experience with any virtual worlds other than SL. The sample was relatively evenly split between Mac (7/13, 54%) and PC users (6/13, 46%).

Of the 13 participating physicians, 3 had previous SL experience. The participants found SL to be an effective method of learning and reported they would like to take other courses in SL. They were mostly neutral or unsure about whether SL is a superior learning technique to face-to-face learning. The majority reported they would recommend SL to a colleague, strongly agreed that SL is effective, and exhibited interest in taking more courses with SL. Interestingly, 10 of the 13 participants reported that engaging in role-play was easier in SL than in face-to-face role-play practice.

### Motivational Interviewing Course Acceptability Outcomes

Acceptability of the MI course content and training was positive overall. The participants rated the MI online tutorial with an average score of 4 (SD 0.9), or “very good,” out of 5,and the lecture on the *empathic partnership* at 4.1(SD 0.86). The *eliciting change talk* lecture was rated similarly, with a mean score of 4.4 (SD 0.63). The participants rated their experiences with mock patients in role-play interviews as well as the performance of their MI coaches. The first mock interview practice session, focusing on *developing empathic partnership*, received an average score of 4.7 (SD 0.63) out of 5 and the second, focusing on *eliciting change talk*, received a mean score of 4.6 (SD 0.87). Participants’ averaged scores for the VASE-R DVD learning tool was 4.1 (SD 1.12).

### Educational Outcomes

Colorectal cancer screening practices and MI skills proficiency changes were measured using survey results and scores from participants’ coded mock interviews of colorectal cancer screening counseling with standardized patients (see Table 1 and Table 2). We examined changes in colorectal cancer screening practice experiences and MI skills proficiency changes. Table 1 shows the results comparing the participants’ pre- and post-training 12-item questionnaire on colorectal cancer screening practice experiences. Following the MI training in SL, the participants reported a significant increase in their knowledge and skills related to colorectal cancer screening counseling. A greater portion (9/13, 69% compared with 5/13, 39%) reported recommending routine colorectal cancer screening to patients over 50 years of age, and 77% (10/13) (compared with 5/13, 38% before training) became “very comfortable” talking to patients about colorectal cancer screenings. In 10 of the 12 survey items, the scores increased with mean differences that are equal to or exceed 1 point on a 7-point Likert scale, which, for the sake of detecting trends in these data, is significant. The participants’ reported ability to address the patients’ barriers to colorectal cancer screening increased significantly—on average, by 1.8 points (see Table 1).

**Table 1:** Participants’ self-reported mean change in knowledge and confidence scores in colorectal cancer screening counseling practices (N = 13 for each item)

	Precourse mean	Postcourse mean	Difference	*P* value
Routine recommendation	5.69	6.23	0.54	.4
Comfortable discussing screening	5.75	6.46	1.08	.03
Effective help coping with concerns	5.15	6.15	1.00	.04
Successful motivation	4.15	5.77	1.62	.004
Effective help overcoming resistance	3.92	5.46	1.54	.007
Knowledge of psychology of decision	4.46	5.67	1.33	.02
Knowledge of health behavior change stages	4.85	6.23	1.38	.02
Communication to promote colorectal cancer screening	4.23	5.77	1.54	.01
Ability to elicit barriers	4.54	6.15	1.61	.004
Ability to address barriers	4.15	6.0	1.85	.004
Detection of ambivalence	4.42	5.85	1.42	.03
Screening follow-up	2.92	4.62	1.69	.007

**Table 2:** Mean pre- to post-training change in global scores for proficiency in motivational interviewing (N = 13 for each component)

	Mean pretraining score	Mean post-training score	Mean change	*P* value
Global	3.05	3.80	0.75	.001
Empathy	3.12	3.85	0.73	.001
Autonomy	3.07	3.85	0.78	<.001
Direction	3.50	3.50	0.00	–
Collaboration	2.88	3.46	0.58	.02
Evocative response	2.80	3.61	0.81	.008

**Figure 3:**
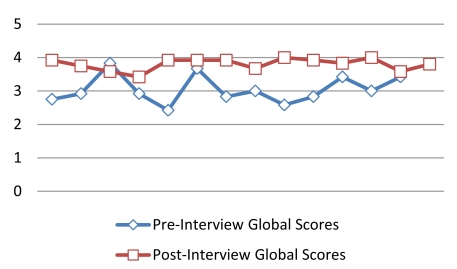
Change in global motivational interviewing scores after training.

In terms of measured MI proficiency, we found significant changes in MI skills based on comparison of audiotaped interview scores before and after MI training in SL (see Table 2 and Figure 3). Before training, only 15% (2/13) of participants received a global clinician score at or above the proficiency level of 3.5 out of a possible 5 points, with a score of 4 indicating MI competency. After the MI training in SL, 12 of the 13 participants scored at or above the MI proficiency level, and 2 participants received scores in the range of MI competency. Group mean scores for the global MI rating and each component score are listed in Table 2. Mean scores for 4 of the 5 component skills increased significantly following MI training (see Table 2): empathy (0.73, *P* = .001), autonomy (0.78, *P* < .001), collaboration (0.58, *P* = .02), and evocative response (0.81, *P* = .008). The evocative response score represents the elements of *eliciting change talk.* Mean global rating scores also increased significantly (0.75, *P* = .001).

## Discussion

Patient-centered communication and counseling skills, such as MI, are a cornerstone of clinically effective health care. Our results indicate that the virtual world holds tremendous promise as a venue for training physicians in these essential skills and improving access to such training for health care professionals. We demonstrated high acceptability and feasibility for conducting training in MI using the SL virtual-world platform. Further, despite a small pilot study sample size, we were able to demonstrate a significant and positive effect on educational outcomes for developing proficiency in global clinician ratings in use of MI and in colorectal cancer screening practices and counseling among 13 primary care providers.

This pilot study supports adapting traditional face-to-face training to virtual-world environments. We were successful in recruiting physicians to participate in this training experience, and each learner rated the experience favorably. Learners acquired the basic navigational skills for SL using a self-directed tutorial with the additional support of a technical skills assessment process that ensured sufficient proficiency in SL before the live learning aspect of the study period began. By using a virtual world, we were able to engage physicians from 6 different states simultaneously in this program. We did find it necessary to have all study staff in one physical location, but technical support personnel were located remotely in 2 different east coast states.

We also achieved excellent results in our limited measure of educational effectiveness in teaching MI in the virtual world. Our study protocol involved a total of 8–10 hours of active learning whereby learners were trained in the philosophy and conceptual model of MI and engaged in role-play for skills development, compared with the traditional 2- to 3-day in-person commitment typically required for essential MI training. We were able to demonstrate a statistically significant improvement in proficiency scores of recorded counseling interviews with a mock patient. While only 2 participants’ scores demonstrate full competency in the global score rating for the MI method overall, we were able to achieve proficiency for 12 of the 13 participants (92%). This suggests that our 8- to 10-hour practical training approach in a virtual world is as effective as a 2- to 3-day in-person training in MI [15]. Finally, our learners reported significant improvements in confidence in colorectal cancer screening counseling after the MI training in SL.

Our training is focused on practical skill-building with a learning-by-doing educational approach. We developed this curriculum based on evidence from the Evaluating Methods for Motivational Enhancement Education clinical trial by Miller et al, indicating the significant impact of feedback and coaching on trainee proficiency in MI [15]. Our findings support this earlier work showing that practice of the MI method combined with coaching and feedback is the most effective approach to clinician skills acquisition. We introduced feedback at two key points in our training, during the SL sessions and between sessions with feedback for participants’ responses on the VASE-R DVD tool. In addition, we provided opportunities for both individualized and group feedback and coaching. This method allowed participants to learn to identify key MI behaviors by observing others and to learn from observing feedback and coaching provided to peer learners, thus optimizing SL participation as an active learning opportunity. Our participants rated this approach highly, indicating the substantial benefit of learning from observing others engaging with the standardized patient. This approach is an adaptation from other MI training methods where feedback and coaching are often done individually.

Our study involved only physicians. It is clear, however, that other health care professionals and nonclinical staff could also benefit from virtual-world training opportunities. Our experience indicates that virtual worlds are amenable to communication skills training beyond health behavior counseling such as discussion about end-of-life and advance directives, communication about medical errors, patient-centered communication for nonclinical staff, and cultural competency training. Some of the unique aspects of a virtual-world training that are conducive to communication skills training are the anonymity of the avatar that facilitates role-play, the ability to simulate a deep, immersive, and realistic experience, and the enhancement of technology skills acquired through technology-based learning environments [23].

While our pilot study demonstrates the enormous potential of virtual-world environments for clinical communication skills training, there are several important limitations. Our study did not correlate the achieved MI skills proficiency with clinical outcomes. Thus, we do not know from our findings whether this level of MI training and achieved proficiency is sufficient to make clinically significant improvements in patient outcomes. Further, it is not evident that this training method is conducive to dissemination on a larger scale. The need for small-group learning and individualized coaching between sessions is certainly a potential limitation to cost effectiveness; however, if learners acquire skills proficiency more quickly with this instructional design, our training approach may prove cost effectiveness. Our next effort will involve a fully powered trial testing the effectiveness of this training to increase colorectal cancer screening rates.

We did experience several SL technical problems during our study period. The problems with using a virtual world like SL for education and training lie mostly in the realm of technical and security issues. The software requires a download and has significant system requirements (processing power, up-to-date video card, and a fast broadband Internet connection), the learning curve for navigation and interaction is steep, and the possibilities for technical problems and failures during the actual event are numerous. Many corporate or university firewalls do not allow access to public virtual worlds like SL.

Despite these limitations, 13 participants successfully completed our study. We learned that it is possible for this participant population to master sufficient virtual-world navigation skills without one-to-one intervention. Recognizing the significant support required for one-to-one training from our prior study, in this study we used a self-directed tutorial to support learners in acquiring SL navigation skills. By doing so, we dramatically reduced the time required for SL skills support, providing only a final skills verification session for each participant. Ultimately, all participants successfully learned the essential skills. On the other hand, the course facilitator was challenged with a high degree of multitasking while teaching. For example, the SL facilitator not only conducted a didactic learning session but also was simultaneously required to monitor the local text chat and respond to questions and comments in real time. In the role-play segment of our training, MI coaches were required to simultaneously navigate the avatar, listen to learners conduct interviews with the mock patient, monitor comments from the local chat of observing learners, and then provide feedback to the interviewer. Managing all of these tasks concurrently occasionally affected the momentum of the training but was not disruptive.

### Conclusion

Overall, the SL virtual-world training in MI appears to have yielded significant increases in the confidence levels of doctors with respect to their abilities to recommend and guide their patients through the colorectal screening procedure and in their proficiency using MI strategies. These results warrant further research in this area, including future work to determine whether this educational approach can be expanded to a larger group of learners in a cost-effective manner and whether the achieved MI proficiency levels correlate with improved patient outcomes.

## Abbreviations

CME: continuing medical education
MI: motivational interviewing
SL: Second Life
VASE-R: video assessment of simulated encounters-revised
